# A Multicenter Retrospective Study Evaluating Intravenous to Oral Antibiotic Stepdown for Uncomplicated Streptococcal Bacteremia

**DOI:** 10.1093/ofid/ofae361

**Published:** 2024-06-28

**Authors:** Alison K Lew, Madison E Salam, Alan E Gross, Sheila K Wang, Erin McGuire, Natasha N Pettit, Jennifer Pisano, Cynthia T Nguyen

**Affiliations:** Department of Pharmacy, University of Chicago Medicine, Chicago, Illinois, USA; Department of Pharmacy, University of Colorado Hospital, Aurora, Colorado, USA; Department of Pharmacy Practice, University of Illinois Chicago College of Pharmacy, Chicago, Illinois, USA; Department of Pharmacy, Northwestern Memorial Hospital, Chicago, Illinois, USA; Department of Pharmacy, Northwestern Memorial Hospital, Chicago, Illinois, USA; Department of Pharmacy, University of Chicago Medicine, Chicago, Illinois, USA; Department of Medicine, University of Chicago Medicine, Chicago, Illinois, USA; Department of Pharmacy, University of Chicago Medicine, Chicago, Illinois, USA

**Keywords:** antimicrobial stewardship, oral stepdown, uncomplicated streptococcal bacteremia

## Abstract

**Background:**

The purpose of this study was to compare the efficacy and safety of intravenous (IV) versus oral (PO) stepdown therapy for uncomplicated streptococcal bacteremia.

**Methods:**

This multicenter, retrospective study included adult patients with uncomplicated streptococcal bacteremia between 1 July 2019 and 1 July 2022. Patients who received IV therapy for the full treatment course were compared to patients who transitioned to PO therapy after initial IV therapy. The primary outcome was clinical success, defined as absence of infection recurrence, infection-related readmission, and infection-related mortality at 90 days. Secondary outcomes included microbiological success, length of stay (LOS), and IV line–associated complications.

**Results:**

Of 238 patients included, 47.1% received PO stepdown therapy. Clinical success occurred in 94.4% and 94.6% in the IV only and PO stepdown groups, respectively (*P* = .946). Patients who transitioned to PO therapy received a median duration of IV therapy of 3.9 days (interquartile range, 2.9–7.3 days). Line complications were more frequent in the IV only group, primarily driven by catheter-related infections (7.2% vs 0%, *P* = .002). LOS was significantly shorter in the PO stepdown group (5.5 vs 9.2 days, *P* < .001).

**Conclusions:**

Patients transitioned to PO antibiotics for uncomplicated streptococcal bacteremia had similar rates of clinical success compared to patients who received only IV therapy. With consideration of infectious source, severity of illness, and comorbidities, PO stepdown following initial IV antibiotics for uncomplicated streptococcal bacteremia in select patients is a reasonable approach that may result in decreased LOS and line-related complications.

Bacteremia is a significant source of morbidity and mortality and is estimated to be the seventh leading cause of death in the United States [1, 2]. Among patients with bacteremia, 30-day mortality has been reported to range from 12% to 24% [3]. Although treatment recommendations for bacteremia vary by pathogen, the traditional treatment involves targeted intravenous (IV) antimicrobial therapy for a duration of 7–14 days [4]. Patients requiring IV antibiotics are predisposed to complications such as secondary catheter-associated infections, thromboses, infiltration, and extravasation. Additionally, IV therapy is associated with a longer duration of hospital stay and higher cost of treatment [5]. These well-recognized risks prompt evaluation of oral (PO) antimicrobials as a stepdown therapy to mitigate adverse effects of parenteral therapy.

Recent studies evaluating PO stepdown therapy for bacteremia caused by Enterobacterales have shown similar rates of mortality and infection recurrence when high-bioavailability antimicrobials are utilized [6–9]. While IV to PO stepdown for uncomplicated gram-negative bacteremia is becoming common practice, there remain limited data utilizing this strategy for uncomplicated streptococcal infections. Streptococci account for almost 10% of community-onset bacteremia with *Streptococcus pneumoniae* being 1 of the 3 most common etiologies of bacteremia [10, 11]. Given the incidence of streptococcal bacteremia, investigating PO antibiotic stepdown may have a significant impact on antimicrobial stewardship practices and minimize adverse sequelae of prolonged IV antibiotics.

The purpose of this study was to compare the efficacy and safety of IV only therapy to PO stepdown therapy for uncomplicated streptococcal bacteremia. We hypothesized that there would be no difference in clinical success for patients who are transitioned from IV to PO antibiotics for stepdown therapy compared to patients who receive IV antibiotics for the full treatment duration.

## METHODS

### Study Subjects

This multicenter, retrospective analysis of patients with streptococcal bacteremia was conducted at 3 academic medical centers (University of Chicago Medicine, Northwestern Memorial Hospital, and University of Illinois Hospital) from 1 July 2019 to 1 July 2022. Approval of the study protocol was obtained from the institutional review board at each study site. The purpose of this study was to compare the clinical success rates of patients with uncomplicated streptococcal bacteremia who received IV antibiotics for the full treatment duration to those who transitioned from IV to PO antibiotics. Adult patients, aged ≥18 years, were included if they had blood cultures positive for *Streptococcus* spp with reported susceptibilities, had ≤48 hours of positive cultures, and received treatment for the bacteremia. Patients were excluded if they had polymicrobial bacteremia, died prior to the completion of treatment, or had a complicated infection. Complicated infection is defined below in “Outcomes and Definitions.”

### Data Collection

Patients were identified using a microbiology report to identify blood cultures positive for *Streptococcus* spp. Manual chart review was utilized to identify eligible candidates for inclusion. All demographic, microbiological, clinical, and follow-up data were collected via retrospective chart review in the electronic health record. Patients were de-identified and data points were recorded into a data collection tool using a secure REDCap database.

### Outcomes and Definitions

The primary outcome was clinical success, defined as the absence of infection recurrence, infection-related readmission, and infection-related mortality at 90 days. Secondary outcomes included individual components of the primary outcome, microbiological success, all-cause readmission at 90 days, all-cause mortality at 90 days, IV line–associated complications at 30 days, antibiotic-associated adverse events (AEs) while on antibiotic therapy, and length of stay (LOS). Infection recurrence was defined as isolation of the same genus and species in the blood within 90 days of first positive blood culture. Infection-related readmission was defined as readmission due to lack of clinical improvement as determined by the treating provider or relapse of the same infection according to either site of infection or organism isolated within 90 days of first positive blood culture. Microbiological success was defined as lack of isolation of infecting organism from any source after initial documented clearance and up to 30 days after completion of therapy. If repeat blood cultures were not obtained, we assumed that microbiological success was achieved. LOS was defined as day 1 of bacteremia until hospital discharge. Line-associated complications and antibiotic-associated AEs were identified using predefined search terms in the electronic health record. Complicated infection was defined as endocarditis and/or endovascular infection, bone and/or joint involvement, device and/or prosthetic involvement, central nervous system involvement, or unattainable source control. Immunocompromised status was defined as neutropenia (absolute neutrophil count <1000 cells/µL), recent chemotherapy within 30 days, treatment with corticosteroids equivalent of prednisone ≥20 mg per day for at least 7 days, human immunodeficiency virus (HIV) infection with a CD4 count <200 cells/µL, solid organ transplant (SOT) recipient, hematopoietic stem cell transplant (HSCT) recipient, or receiving other immunosuppressive medications. The Pitt Bacteremia Score was used to assess the severity of acute illness [12]. Moderate and severe liver and renal disease were defined by criteria outlined in the Charlson Comorbidity Index [13]. Moderate liver disease was defined as cirrhosis with portal hypertension without bleeding, and severe liver disease was defined as cirrhosis, portal hypertension, and history of variceal bleeding. Moderate renal disease was defined as serum creatinine >3 mg/dL, and severe renal disease was defined as requirement of dialysis, receipt of renal transplant, and presence of uremia.

### Data Analysis

Statistical analyses were performed utilizing χ^2^ test or Fisher exact test for categorical variables and Student *t* test or Mann-Whitney *U* test for continuous variables as appropriate. Estimating clinical success rates from other studies that evaluated PO stepdown for uncomplicated streptococcal bacteremia [14–17], we estimated 90% success in both groups. Using an α of .05 and β of .2, the sample size required to reach 80% power was calculated to be 224 patients with 112 patients in each group. Logistic regression was used to estimate the odds ratios (ORs) and 90% confidence intervals (CIs) for categorical outcomes and linear regression was used to estimate the ORs and 90% CIs for continuous outcomes. Multiple regression analysis was used to estimate the ORs and 90% CIs for the primary outcome. The use of PO stepdown and variables that differed between the primary outcome subgroups in univariate analysis (*P* < .1) were then included in the multiple regression analysis to identify independent predictors of the primary outcome. All statistical analyses were performed using Stata software (version 16.1, StataCorp LLC, College Station, Texas).

## RESULTS

**Figure 1.**
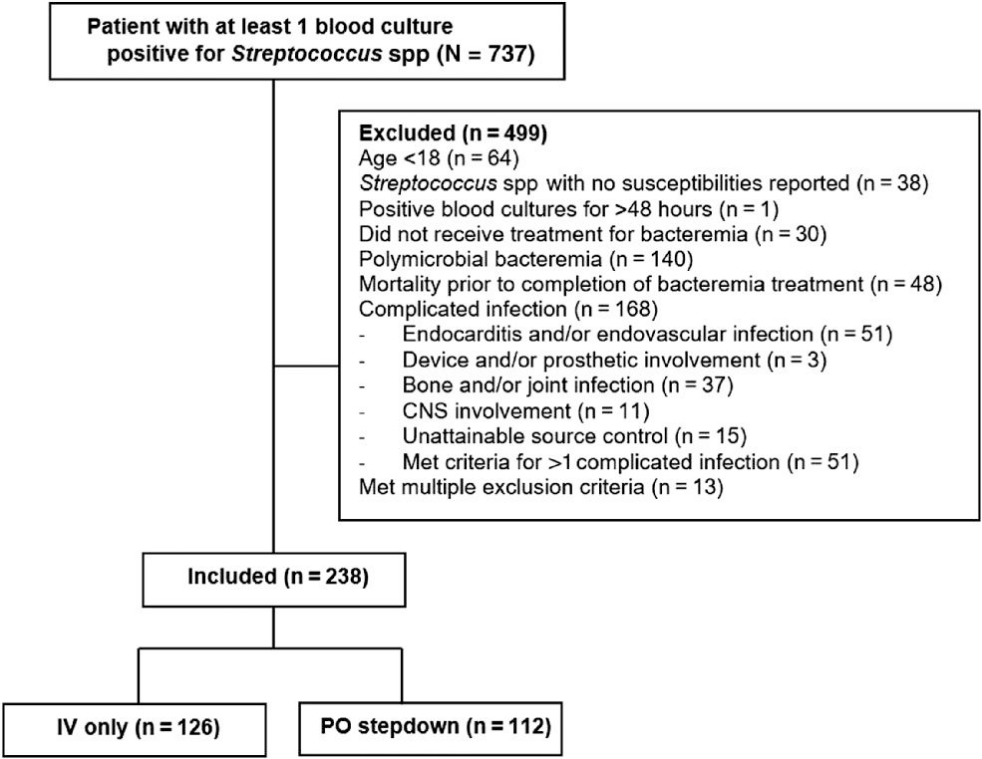
Inclusion and exclusion criteria. Abbreviations: CNS, central nervous system; IV, intravenous; PO, oral.

Among the 737 patients identified as having a positive blood culture for a *Streptococcus* spp, 499 patients were excluded. Reasons for exclusion are outlined in Figure 1. Of the 238 patients included in the final analysis, 126 (52.9%) patients received IV antibiotics for the full treatment course while 112 (47.1%) were transitioned from IV to PO therapy. Baseline characteristics were similar between the 2 groups, with an overall median age of 59.5 years, a median Pitt Bacteremia Score of 1 (interquartile range [IQR], 0–2), and Charlson Comorbidity Index score of 4 (IQR, 2–6). Neutropenia (34.9% vs 17%, *P* = .002), recent chemotherapy within 30 days (38.1% vs 22.3%, *P* = .008), and HSCT (23.8% vs 13.4%, *P* = .041) were more common in the IV group. HIV infection (6.4% vs 0%, *P* = .005) was more common in the PO stepdown group. There were similar rates of patients receiving IV only and PO stepdown among SOT recipients and patients who received steroids or other immunosuppressive medications (Table 1).

**Table 1.** Baseline Characteristics of Included Patients

Characteristic	Overall (n = 238)	IV Only (n = 126)	PO Stepdown (n = 112)	*P* Value
Age, y, median (IQR)	59.5 (47–68)	61 (51–68)	56 (45–68)	.254
Sex, male	116 (48.7)	59 (46.8)	57 (50.9)	.531
BMI, kg/m^2^, median (IQR)	26.9 (22.9–33.4)	26.6 (22.5–34.2)	27.4 (23.7–33.2)	.607
ICU admission	43 (18.1)	28 (22.2)	15 (13.4)	.077
Pitt Bacteremia Score, median (IQR)	1 (0–2)	1 (0–2)	1 (0–2)	.369
ID consult	176 (74.0)	98 (77.8)	78 (69.6)	.154
CCI score, median (IQR)	4 (2–6)	4 (3–6)	4 (2–6)	.297
Comorbidities				
Diabetes	89 (37.4)	46 (36.5)	43 (38.4)	.764
Moderate or severe renal disease	44 (18.5)	27 (21.4)	17 (15.2)	.215
Moderate or severe liver disease	13 (5.5)	7 (5.6)	6 (5.4)	.946
Cerebrovascular disease	15 (6.3)	9 (7.1)	6 (5.4)	.571
Hemiplegia	3 (1.3)	1 (0.8)	2 (1.8)	.602
Immunocompromised	118 (49.6)	69 (54.8)	49 (43.8)	.090
Absolute neutrophil count <1000/mL	63 (26.5)	44 (34.9)	19 (17.0)	.002
Recent chemotherapy within 30 d	73 (30.7)	48 (38.1)	25 (22.3)	.008
Solid organ transplant	15 (6.3)	10 (7.9)	5 (4.5)	.299
Hematopoietic stem cell transplant	45 (18.9)	30 (23.8)	15 (13.4)	.041
HIV infection	7 (2.9)	0 (0.0)	7 (6.3)	.005
Receipt of steroids or other immunosuppressive medications	41 (17.2)	24 (19.1)	17 (15.2)	.430
Source of bacteremia				
Skin/soft tissue	60 (25.2)	28 (22.2)	32 (28.6)	.260
Catheter-related	35 (14.7)	26 (20.6)	9 (8.0)	.006
Intra-abdominal	33 (13.9)	20 (15.9)	13 (11.6)	.342
Respiratory	30 (12.6)	10 (7.9)	20 (17.9)	.021
Oropharyngeal	22 (9.2)	13 (10.3)	9 (8.0)	.544
Genitourinary	15 (6.3)	3 (2.4)	12 (10.7)	.014
Sinusitis	4 (1.7)	2 (1.6)	2 (1.8)	>.999
Unknown	34 (14.3)	22 (17.5)	12 (10.7)	.138
Other^a^	5 (2.1)	2 (1.6)	3 (2.7)	.668
Causative pathogen				
*Streptococcus mitis*	70 (29.4)	48 (38.1)	22 (19.6)	.002
*Streptococcus agalactiae*	37 (15.6)	18 (14.3)	19 (17.0)	.569
*Streptococcus pneumoniae*	34 (14.3)	12 (9.5)	22 (19.6)	.026
*Streptococcus pyogenes*	28 (11.8)	11 (8.7)	17 (15.2)	.123
*Streptococcus dysgalactiae*	15 (6.3)	6 (4.8)	9 (8.0)	.300
*Streptococcus anginosus*	8 (3.4)	5 (4.0)	3 (2.7)	.726
*Streptococcus salivarius*	5 (2.1)	4 (3.2)	1 (0.9)	.374
Other *Streptococcus* spp	41 (17.2)	22 (17.5)	19 (17.0)	.919
Time to active antibiotics, h, median (IQR)	1.9 (0.2–5.5)	1.5 (−0.4 to 5.9)	2 (0.5–4.9)	.312

Data are presented as No. (%) unless otherwise indicated.

Abbreviations: BMI, body mass index; CCI, Charlson Comorbidity Index; HIV, human immunodeficiency virus; ICU, intensive care unit; ID, infectious diseases; IQR, interquartile range; IV, intravenous; PO, oral.

^a^Other sources of bacteremia included 2 surgical site infections, 1 G-tube site infection, 1 ischiorectal abscess, and 1 supraclavicular abscess.

The most common infectious sources were skin and soft tissue (25.2%), catheter-related (14.7%), intra-abdominal (13.9%), and respiratory (12.6%). Among patients with an intra-abdominal source, 75.8% were classified as gut translocation. Of the remaining 24.2% of patients with intra-abdominal sources, surgical intervention was performed in only 75% of all cases in which surgical intervention was warranted. Patients were more likely to receive PO stepdown if they had a respiratory source (17.9% vs 7.9%, *P* = .021) or genitourinary source (10.7% vs 2.4%, *P* = .014), whereas patients with a catheter-related source were more likely to remain on IV antibiotics for the full duration (20.6% vs 8.0%, *P* = .006).

The most common pathogens isolated were *Streptococcus mitis* (29.4%), *Streptococcus agalactiae* (15.6%), and *S pneumoniae* (14.3%) (Table 1). More patients with *S mitis* bacteremia received IV therapy for the full duration (38.1% vs 19.6%, *P* = .002), whereas more patients with *S pneumoniae* received PO stepdown therapy (19.6% vs 9.5%, *P* = .026). Of the patients who had *S mitis* bacteremia, the source of bacteremia was identified to be catheter-related or had an unknown source in 55.7% of patients. Of patients who had *S pneumoniae* bacteremia, the most common source was identified to be respiratory (64.7%).

Time to active antibiotics in hours from positive blood culture to antibiotic initiation was similar between the IV only and PO stepdown groups (median hours, 1.5 [IQR, −0.4 to 5.9] vs 2 [IQR, 0.5–4.9]; *P* = .312). Among patients who transitioned to PO therapy, the median duration of IV therapy was 3.9 days (IQR, 2.9–7.3). The majority of patients in the PO stepdown group were transitioned to a PO β-lactam (69.6%). The most common antibiotics for PO stepdown included amoxicillin-clavulanate (26.8%), levofloxacin (21.4%), and cefdinir (14.3%) (Table 2). The most common antibiotics for definitive IV therapy included ceftriaxone (69.8%), vancomycin (12.7%), and cefepime (7.1%). Total antibiotic durations were similar between the IV only and PO stepdown group (median days, 13.5 [IQR, 11.5–14.6] vs 13.3 [IQR, 12.2–15.1]; *P* = .649).

**Table 2.** Oral Stepdown Regimens and Dosing

Antibiotic	Dose	Frequency, No. (%)
Amoxicillin	Total	15 (13.4)
	500 mg q12h	1 (0.9)
	500 mg q8h	3 (2.7)
	1000 mg q12h	1 (0.9)
	1000 mg q8h	10 (8.9)
Amoxicillin/clavulanate	875 mg q12h	30 (26.8)
Cephalexin	Total	14 (12.5)
	500 mg q12h	1 (0.9)
	500 mg q6h	5 (4.5)
	1000 mg q8h	8 (7.1)
Cefdinir	300 mg q12h	16 (14.3)
Cefpodoxime	400 mg q12h	2 (1.8)
Cefixime	400 mg q24h	1 (0.9)
Levofloxacin	Total	24 (21.4)
	500 mg q24h	3 (2.7)
	750 mg q24h	21 (18.8)

Patients with renal impairment at the time of oral antibiotic initiation received appropriate renal dose adjustment. The dosing regimen listed represents the equivalent dose normalized for renal impairment. The remaining oral antibiotics not characterized in this table were clindamycin (4 [3.5%]), linezolid (3 [2.7%]), moxifloxacin (2 [1.8%]), and doxycycline (1 [0.9%]).

The composite outcome of clinical success occurred in 94.4% of patients in the IV only group and 94.6% of patients in the PO stepdown group (*P* = .946; Table 3). Infection recurrence, infection-related readmission, and infection-related mortality were similar between the IV only and PO stepdown group. Multiple regression analysis was performed and only *Streptococcus anginosus* (OR, 0.132 [90% CI, .027–.642]; *P* = .03) and other *Streptococcus* spp (OR, 0.248 [90% CI, .084–.728]; *P* = .03) as causative pathogens were associated with a decreased odds of clinical success (Supplementary Table 2). In total, 13 patients experienced clinical failure where 1 patient experienced both infection recurrence and infection-related readmission. Details regarding the reasons for clinical failure are outlined in Supplementary Table 1.

**Table 3.** Clinical Outcomes

Outcome	Overall (n = 238)	IV (n = 126)	IV to PO (n = 112)	OR (90% CI)	*P* Value
Clinical success	225 (94.5)	119 (94.4)	106 (94.6)	1 (.44–2.29)	.946
Reasons for clinical failure					
Infection recurrence	2 (0.8)	2 (1.6)	0 (0)	…	.500
Infection-related mortality	3 (1.3)	2 (1.6)	1 (0.9)	0.56 (.07–4.24)	.636
Infection-related readmission	9 (3.8)	4 (3.2)	5 (4.5)	1.33 (.52–3.42)	.738
All-cause mortality	13 (5.5)	6 (4.8)	7 (6.3)	1.33 (.52–3.42)	.615
All-cause readmission	94 (39.5)	53 (42.1)	41 (36.6)	0.86 (.55–1.33)	.390
Antibiotic- or line-related readmission	4 (1.7)	4 (3.2)	0 (0)	…	.124
Antibiotic-related readmission	0 (0)	0 (0)	0 (0)	…	>.999
Line-related readmission	4 (1.7)	4 (3.2)	0 (0)	…	.124
Microbiological success	234 (98.3)	124 (98.4)	110 (98.2)	0.89 (.17–4.66)	.905
Line complications	10 (4.2)	10 (7.9)	0 (0)	…	.002
Catheter-related infection	9 (3.8)	9 (7.1)	0 (0)	…	.004
Catheter-related thrombosis	0 (0)	0 (0)	0 (0)	…	>.999
Extravasation	1 (0.4)	1 (0.8)	0 (0)	…	>.999
Antibiotic-associated AE while on antibiotic therapy	14 (5.9)	6 (4.8)	8 (7.1)	1.54 (.62–3.84)	.439
Antibiotic-associated diarrhea	8 (3.4)	3 (2.4)	5 (4.5)	1.92 (.57–6.49)	.381
Antibiotic-associated GI symptoms	4 (1.7)	1 (0.8)	3 (2.7)	3.44 (.51–23.27)	.288
Antibiotic hypersensitivity reaction	6 (2.5)	3 (2.4)	3 (2.7)	1.13 (.29–4.40)	.884
Antibiotic duration, d, median (IQR)	13.4 (12.1–14.7)	13.5 (11.5–14.6)	13.3 (12.2–15.1)	0.28 (−.74 to 1.31)	.649
IV antibiotic duration, d, median (IQR)	11.1 (5–13.7)	13.5 (11.2–14.4)	3.9 (2.9–7.3)	−8.99 (−10.85 to −7.14)	<.001
Length of stay, d, median (IQR)	7.4 (4.6–11.8)	9.2 (6.2–16.2)	5.5 (3.5–8.3)	−5.97 (−9.35 to −5.30)	<.001

Data are presented as No. (%) unless otherwise indicated.

Abbreviations: AE, adverse event; CI, confidence interval; GI, gastrointestinal; IQR, interquartile range; IV, intravenous; OR, odds ratio; PO, oral.

There were more line complications in the IV only group (7.9% vs 0%, *P* = .002), primarily driven by catheter-related infections (7.1% vs 0%, *P* = .004) as there were no catheter-related thromboses and only 1 case of extravasation in the IV only group. More patients had line-related readmissions in the IV only group, but this was not statistically significant (3.2% vs 0%, *P* = .124). Other antibiotic-associated AEs while on antibiotic therapy were similar between the IV only and PO stepdown group (4.8% vs 7.1%, *P* = .439). LOS was significantly shorter in the PO stepdown group (median days, 5.5 [IQR, 3.5–8.3] vs 9.2 [IQR, 6.2–16.2]; *P* < .001; Table 3).

## DISCUSSION

In this multicenter study, patients treated for uncomplicated streptococcal bacteremia with IV antibiotics for the full duration and those who received PO stepdown after initial IV antibiotics experienced similar clinical success rates. Our observed clinical success rates are consistent with those reported among patients treated for uncomplicated streptococcal bacteremia [14–17]. Two single-center retrospective studies demonstrated similar rates of infection recurrence, readmission, and mortality among patients who received PO stepdown or continued IV therapy for uncomplicated streptococcal bacteremia [15, 17]. We observed a median of 3.9 days of IV therapy in patients who received PO stepdown, consistent with previously published studies. In the study by Kang et al, transition from IV to PO therapy occurred at a median of 4 days; in the study by Waked et al, patients were excluded from the study if they were transitioned to PO antibiotic therapy >5 days after a positive blood culture [15, 17]. Ramos-Otero et al reported an average of 4.4 days of IV therapy among the IV to PO group with no association of worse clinical outcomes compared to their IV only group [16]. Together, these data support early transition to PO antibiotic therapy for the completion of uncomplicated streptococcal bacteremia treatment.

Most (69.7%) of our PO stepdown regimens were comprised of β-lactam antibiotics, with amoxicillin and amoxicillin-clavulanate making up 40.2% of all PO stepdown regimens. Amoxicillin and amoxicillin-clavulanate comprised 55.6%, 39%, and 22% of all PO stepdown regimens in the studies by Arensman et al, Waked et al, and Kang et al, respectively [14, 15, 17]. While cefdinir and cephalexin were infrequently prescribed in the studies by Arensman et al and Kang et al, 25% and 17% of PO stepdown regimens in the study by Waked et al consisted of cefdinir and cephalexin, respectively. In contrast, cefdinir was prescribed in 14.3% and cephalexin was prescribed in 12.5% of patients in the PO stepdown group of our study. Last, use of levofloxacin for PO stepdown was comparable in our study to that in the study by Waked et al (21.4% vs 17%), which is remarkably lower than that observed in the studies by Arensman et al and Kang et al, which were 58.6% and 40%, respectively [14, 15, 17]. The heterogeneity of PO stepdown regimens among the various studies makes it difficult to directly compare outcomes based on the PO antibiotics received. However, β-lactams may be preferred over fluoroquinolones for PO stepdown based on similar outcomes observed and the known advantages of minimizing unnecessary fluoroquinolone use.

There are a few notable differences between this study and the studies discussed. First, this was a multicenter study, whereas the prior studies were single-center studies. The present study included patients from 3 academic medical centers in Chicago, Illinois. This allowed us to capture a more diverse patient population that may increase the generalizability of these results among patients meeting criteria for uncomplicated streptococcal bacteremia. Second, we performed a power analysis and achieved the necessary sample size to meet power, unlike prior studies. We additionally performed a multiple regression analysis to identify independent predictors of our primary outcome, clinical success, which previous studies did not. Third, approximately half of the patients in this study were immunocompromised, whereas prior studies had relatively low inclusion of immunocompromised hosts. SOT and HSCT recipients comprised 25% of all included patients. The studies by Kang et al and Ramos-Otero et al did not report any patients with a history of transplant, while Waked et al only reported 6% of all patients with a history of transplant [15–17]. Last, the 3 prior studies showed that patients in the IV to PO group had a longer total duration of antibiotic therapy compared to patients in the IV group while this study showed no difference in total antibiotic duration between the 2 groups.

A potential perceived barrier to PO stepdown therapy is the bioavailability concern with PO β-lactams. However, published pharmacokinetic/pharmacodynamic (PD) analyses have found a high probability of target attainment with certain PO β-lactams for streptococcal bacteremia when dosed appropriately. Mogle et al summarized the probability of PD target attainment with various PO β-lactams, taking into consideration the necessary percentage of time above the minimum inhibitory concentration (MIC) of the dosing interval at various doses and intervals and at varying MICs [18]. Importantly, they evaluate PD target attainment with consideration of the wild-type MIC values occurring at the highest frequency for *Escherichia coli*, which ranged from 0.5 mg/L to 4 mg/L. For *E coli*, the wild-type MICs occurring in the highest frequency were 4 mg/L for amoxicillin, 4 mg/L for cephalexin, and 0.5 for cefpodoxime. Applying this same principle for *S pneumoniae*, the wild-type MICs occurring in the highest frequency were 0.016 mg/L for amoxicillin, 1 mg/L for cephalexin, and 0.03 mg/L for cefpodoxime [19]. While Mogle et al conclude that cefdinir appears to offer low likelihood of PD target attainment for Enterobacterales, this is likely not the case for streptococci where the wild-type MICs occurring in the highest frequency is 0.06 mg/L for *S pneumoniae* [18, 19]. Bock et al demonstrated in their POET substudy 100% probability of target attainment in relation to both clinical breakpoints and MICs for streptococci using amoxicillin 1000 mg PO every 6 hours [20, 21]. While dose optimization of PO β-lactams remains an important consideration for the treatment of bacteremia, the considerably lower MICs for streptococci support high likelihood of PD target attainment of PO β-lactams for streptococcal bacteremia at commonly used doses (Table 2). Therefore, in clinical practice, β-lactams with low PO bioavailability such as cefdinir and cefpodoxime at standard doses and highly bioavailable β-lactams such as amoxicillin and cephalexin at lower doses may be reasonable therapeutic options for patients with uncomplicated streptococcal bacteremia despite valid concerns regarding their role in the treatment of uncomplicated gram-negative bacteremia.

In our study, patients who had neutropenia, received chemotherapy within 30 days, and had a history of HSCT were more likely to receive IV therapy for the full duration. A potential explanation for the higher rate of IV therapy in this patient population is the presence of mucositis where reliable gut absorption of PO antibiotics may be of concern. An alternative explanation for higher rates of IV antibiotics may be due to the higher likelihood of this cohort having IV access for reasons unrelated to IV antibiotic administration in conjunction with clinicians’ reservations with transition to PO antibiotics in this vulnerable patient population. Patients with a respiratory source and *S pneumoniae* as a bloodstream pathogen were both more likely to receive PO stepdown. Given that *S pneumoniae* is a common respiratory pathogen, it is likely that clinicians were comfortable in the diagnosis of an uncomplicated infection, and therefore PO stepdown, when the pathogen and source of infection were clear-cut.

In the multiple regression analysis, only *S anginosus* and other *Streptococcus* spp were associated with decreased odds of clinical success, whereas PO stepdown and other patient demographics and factors did not affect clinical success. This finding may be due to the propensity for *S anginosus* to cause abscesses, which can lead to recurrent infection in the setting of inadequate source control. All 3 cases of infection-related mortality were unrelated to the index infection. Of the 9 cases of infection-related readmission, 4 were thought to be unrelated to the index infection while the remaining 5 were likely related to the index infection. Three of the 5 infection-related readmissions were attributable to inadequate source control and 1 was attributable to inadequate initial treatment with clindamycin that was intermediate to the index *Streptococcus* isolated in blood culture.

Several limitations of this study are worth noting. First, this study is retrospective, which predisposes the study to selection bias, as complicated patients may have been more likely to receive IV only therapy. While many baseline characteristics were similar between the 2 groups, there were imbalances among patients with immunocompromising conditions and varying sources of infection, as previously discussed. These differences may limit the ability to extrapolate safety and efficacy of PO stepdown among a select subset of immunocompromised hosts (eg, neutropenia, recent chemotherapy, and HSCT) and in catheter-related infections. Second, 5.6% of excluded patients had *Streptococcus* spp with no susceptibilities reported. While the majority (92.7%) of susceptibilities were not reported because susceptibilities were not automatically performed when only 1 of 2 sets of blood cultures was positive, these data only support PO stepdown for *Streptococcus* spp where susceptibilities can be confirmed. Third, we did not collect MIC data and therefore cannot specifically correlate MIC data with PO antibiotics prescribed. However, the European Committee on Antimicrobial Susceptibility Testing database confirms low β-lactam MICs for streptococcal isolates based on MIC distributions for various *Streptococcus* spp. Fourth, there was no minimum amount of time that a patient had to be on PO antibiotics in order to be assigned to the PO group if they received any duration of PO antibiotics. The duration of therapy for streptococcal bacteremia, as well as the duration of IV lead-in, is not well defined, so we may be overtreating with IV and/or PO antibiotics. Fifth, missing data may have skewed the results. Readmissions and AEs occurring at outside facilities may not have been captured in this study. AEs were only captured if documented in the medical record, so the possibility of missing AEs that were not documented is a limitation. Repeat blood cultures may not have been obtained, which may have inflated the rates of microbiologic success. Additionally, adherence to outpatient antibiotics was not assessed after discharge. Sixth, patients who died prior to completion of therapy were excluded, which may have increased survivor bias and selected for a lower-risk population. We believe that patients who died prior to completion of antibiotic therapy would have been unlikely to meet inclusion criteria for uncomplicated streptococcal bacteremia or to achieve satisfactory clinical response to initial treatment necessary for consideration of PO stepdown; therefore, we are not concerned that excluding these patients would have confounded our dataset. Finally, only 238 of 737 patients screened over the study period met eligibility criteria due to the strict criteria for uncomplicated bacteremia. Our study findings are not generalizable to patients with complicated infections, and scrutiny of the patients included should be taken into account when interpreting the results.

## CONCLUSIONS

This study demonstrated similar rates of clinical success among patients with uncomplicated streptococcal bacteremia who continued IV therapy for the full duration of treatment compared to those who received IV to PO stepdown therapy. PO stepdown therapy was associated with decreased LOS and line complications in comparison to the IV only group. With consideration of infectious source, severity of illness, and comorbidities, PO stepdown after initial IV antibiotics for uncomplicated streptococcal bacteremia in select patients is a reasonable approach that may result in decreased LOS and line complications.

## Supplementary Material

ofae361_Supplementary_Data

## Supplementary Data

Supplementary materials are available at *Open Forum Infectious Diseases* online. Consisting of data provided by the authors to benefit the reader, the posted materials are not copyedited and are the sole responsibility of the authors, so questions or comments should be addressed to the corresponding author.

## Notes

***Acknowledgments*.** The authors would like to acknowledge Hoang Tran, PharmD, Anh Nguyen Quynh Dam, and Joann Huynh, PharmD for their assistance with data collection.

***Patient consent.*** The study protocol was approved by the institutional review board at each study site.
