# Supplementary material for: A Multicenter Retrospective Study Evaluating Intravenous to Oral Antibiotic Stepdown for Uncomplicated Streptococcal Bacteremia
Source: Open Forum Infect Dis. 2024 Jun 28;11(7):ofae361. doi: 10.1093/ofid/ofae361 (PMC11227220; doi:10.1093/ofid/ofae361)
Supplement: ofae361_Supplementary_Data [file ofae361_supplementary_data.docx]

**Table S1. Reasons for Clinical Failure**

| **Reasons for Clinical Failure**  **N=13** | ***Streptococcus* spp.** | **Initial source of infection** | **Antibiotic course** | **Case details regarding clinical failure** |
| --- | --- | --- | --- | --- |
| Infection Recurrence and Infection-related Readmission N = 1 | *S. parasanguinis* | Gut translocation from biliary source | Vancomycin (IV only) | Infection-related readmission 34 days after antibiotic treatment completed with blood cultures positive 21 days into readmission. Same organism isolated in blood culture secondary to gut translocation with concern for inadequate source control in a patient with underlying malignancy. |
| Infection Recurrence N=1 | *S. mitis* | Skin and soft tissue infection | Vancomycin (IV only) | Infection recurrence occurred 23 days after treatment completed. Same *S. mitis* isolated in blood culture thought to be from a catheter-related source rather than skin and soft tissue source from index infection. |
| Infection-related Mortality N=3 | *S. anginosus* | Unknown | Ceftriaxone (IV only) | Infection-related mortality occurred 23 days after antibiotic treatment completed unrelated to the index infection. Infectious course complicated by SARS-COV-2 pneumonia and Pseudomonal pneumonia with respiratory failure requiring intubation and septic shock with subsequent negative blood cultures. |
|  | *S. mitis* | Catheter-related | Ceftriaxone (IV only) | Infection related mortality occurred 44 days after treatment completed due to an unrelated infection causing sepsis and multi-organ failure with *S. epidermidis* isolated in blood culture. |
|  | Viridans Group *Streptococcus* | Unknown | Ceftriaxone followed by PO stepdown to linezolid | Infection-related mortality occurred 43 days after treatment completed due to an unrelated infection with *Pseudomonas* and *Enterococcus* isolated from a respiratory culture. |
| Infection-related Readmission N=8 | *S. anginosus* | Intra-abdominal infection | Ampicillin-sulbactam followed by PO stepdown to amoxicillin-clavulanate | Infection-related readmission was due to relapse of the same infection according to site of infection likely related to index infection. Relapse was likely secondary to a source control issue. Appendicitis during the index infection was medically managed without surgical intervention requiring readmission for management of ruptured appendicitis with concern for early abscess formation. |
|  | *S. agalactiae* | Skin and soft tissue infection | Ceftriaxone followed by PO stepdown to cephalexin | Infection-related readmission was due to relapse of the same infection according to site of infection likely unrelated to index infection. The index infection was due to a left foot abscess whereas the subsequent readmission was related to right foot abscess and osteomyelitis with associated MRSA bacteremia. |
|  | *S. agalactiae* | Skin and soft tissue infection | Ceftriaxone followed by PO stepdown to clindamycin | Infection-related readmission was due to relapse of the same infection according to site of infection likely related to index infection. Relapse was likely secondary to suboptimal treatment with clindamycin as PO stepdown during the index infection. At the time of initial infection, the *S. agalactiae* in blood culture was intermediate to clindamycin. Inadequate source control may have also contributed to relapse as patient required repeat incision and drainage during readmission. |
|  | *S. agalactiae* | Skin and soft tissue infection | Penicillin (IV only) | Infection-related readmission was due to relapse of the same infection according to site of infection likely unrelated to index infection.  The index infection was related to necrotizing fasciitis of the lower extremity while readmission was due to increased wound drainage of the right hip concerning for abscess or infected hematoma. The subsequent SSTI was unrelated with respect to site of infection as well as pathogens isolated in wound cultures, which grew *Enterobacter cloacae*, *Klebsiella pneumoniae*, and *Enterococcus faecium*. |
|  | *S. pyogenes* | Skin and soft tissue infection | Vancomycin followed by PO stepdown to amoxicillin-clavulanate | Infection-related readmission was due to relapse of the same infection according to site of infection likely unrelated to index infection. Relapse likely secondary to inadequate source control of right lower extremity with progression to osteomyelitis and septic arthritis with different pathogens growing in wound cultures (*Enterobacter cloacae* and coagulase negative *Staphylococcus*) than index infection (S*. pyogenes* in both blood and wound culture). |
|  | *S. constellatus* | Sinusitis | Cefepime and vancomycin followed by PO stepdown to amoxicillin-clavulanate | Infection-related readmission was due to relapse of the same infection according to site of infection likely related to index infection. Relapse was likely secondary to a source control issue requiring endoscopic sinus surgery. |
|  | *S. parasanguinis* | Intra-abdominal infection | Vancomycin (IV only) | Infection-related readmission was due to relapse of the same infection according to site of infection likely unrelated to index infection. Relapse likely related to a source control issue in the setting of an infected hepatic biloma with recent ERCP and stent placement. |
|  | *Streptococcus mitis/oralis* group | Intra-abdominal infection | Vancomycin (IV only) | Infection-related readmission was due to relapse of the same infection according to organism isolated in blood culture likely related to the index infection. Repeat positive blood cultures grew the same index organism with *Streptococcus* *mitis/oralis* group for which the source may have been gut translocation as initially presumed, however source of infection during the readmission remained unclear. |

**Table S2. Patient Demographics and Factors Associated with Clinical Success in Patients Treated for Uncomplicated Streptococcal Bacteremia**

|  | **Overall**  **(n=238)** | **Clinical failure**  **(n=13)** | **Clinical success**  **(n=225)** | **Univariate analysis**  **p-value** | **Multivariate analysis odds ratio (90% CI)** |
| --- | --- | --- | --- | --- | --- |
| Age (Median, IQR) | 59.5 (47, 68) | 55 (50, 61) | 60 (47, 68) | 0.243 |  |
| Male (n, %) | 116 (48.7) | 7 (53.8) | 109 (48.4) | 0.705 |  |
| BMI (Median, IQR) | 26.9 (22.9, 33.4) | 27.5 (26.4, 31.9) | 26.6 (22.9, 33.8) | 0.662 |  |
| ICU admission (n, %) | 43 (18.1) | 1 (7.7) | 42 (18.7) | 0.473 |  |
| Pitt Bacteremia Score (Median, IQR) | 1 (0, 2) | 1 (0, 2) | 1 (0, 2) | 0.722 |  |
| ID consult (n, %) | 176 (74.0) | 8 (61.5) | 168 (74.7) | 0.332 |  |
| IV to PO stepdown (n, %) | 112 (47.1) | 6 (46.2) | 106 (47.1) | >0.999 | NS |
| Charlson Comorbidity Index (Median, IQR) | 4 (2, 6) | 3 (2, 5) | 4 (3, 6) | 0.271 |  |
| Comorbidities (n, %) |  |  |  |  |  |
| Diabetes (n, %) | 89 (37.4) | 8 (61.5) | 81 (36.0) | 0.080 | NS |
| Moderate or severe renal disease (n, %) | 44 (18.5) | 1 (7.7) | 43 (19.1) | 0.472 |  |
| Moderate or severe liver disease (n, %) | 13 (5.5) | 2 (15.4) | 11 (4.9) | 0.153 |  |
| Cerebrovascular disease (n, %) | 15 (6.3) | 2 (15.4) | 13 (5.8) | 0.193 |  |
| Hemiplegia (n, %) | 3 (1.3) | 0 (0) | 3 (1.3) | >0.999 |  |
| Immunocompromised (n, %) | 118 (49.6) | 6 (46.2) | 112 (49.8) | 0.799 |  |
| Absolute neutrophil count <1000/ml (n, %) | 63 (26.5) | 4 (30.8) | 59 (26.2) | 0.749 |  |
| Recent chemotherapy within 30 days (n, %) | 73 (30.7) | 2 (15.4) | 71 (31.6) | 0.354 |  |
| Solid organ transplant (n, %) | 15 (6.3) | 2 (15.4) | 13 (5.8) | 0.193 |  |
| Hematopoietic stem cell transplant (n, %) | 45 (18.9) | 2 (15.4) | 43 (19.1) | >0.999 |  |
| HIV infection (n, %) | 7 (2.9) | 0 (0) | 7 (3.1) | >0.999 |  |
| Receipt of steroids or other immunosuppressive medications (n, %) | 41 (17.2) | 4 (30.8) | 37 (16.4) | 0.247 |  |
| Source of bacteremia (n, %) |  |  |  |  |  |
| Skin/Soft Tissue (n, %) | 60 (25.2) | 5 (38.5) | 55 (24.4) | 0.322 |  |
| Catheter-Related (n, %) | 35 (14.7) | 1 (7.7) | 34 (15.1) | 0.698 |  |
| Intra-abdominal (n, %) | 33 (13.9) | 4 (30.8) | 29 (12.9) | 0.088 | NS |
| Respiratory (n, %) | 30 (12.6) | 0 (0) | 30 (13.3) | 0.381 |  |
| Oropharyngeal (n, %) | 22 (9.2) | 0 (0) | 22 (9.8) | 0.616 |  |
| Genitourinary (n, %) | 15 (6.3) | 0 (0) | 15 (6.7) | >0.999 |  |
| Sinusitis (n, %) | 4 (1.7) | 1 (7.7) | 3 (1.3) | 0.202 |  |
| Unknown (n, %) | 34 (14.3) | 2 (15.4) | 32 (14.2) | >0.999 |  |
| Other (n, %) | 5 (2.1) | 0 (0) | 5 (2.2) | >0.999 |  |
| Causative Pathogen (n, %) |  |  |  |  |  |
| *S. mitis* (n, %) | 70 (29.4) | 2 (15.4) | 68 (30.2) | 0.355 |  |
| *S. agalactiae* (n, %) | 37 (15.6) | 3 (23.1) | 34 (15.1) | 0.432 |  |
| *S.* *pneumoniae* (n, %) | 34 (14.3) | 0 (0) | 34 (15.1) | 0.224 |  |
| *S. pyogenes* (n, %) | 28 (11.8) | 1 (7.7) | 27 (12.0) | >0.999 |  |
| *S. dysgalactiae* (n, %) | 15 (6.3) | 0 (0) | 15 (6.7) | >0.999 |  |
| *S. anginosus* (n, %) | 8 (3.4) | 2 (15.4) | 6 (2.7) | 0.064 | 0.132 (0.027, 0.642) |
| *S. salivarius* (n, %) | 5 (2.1) | 0 (0) | 5 (2.2) | >0.999 |  |
| Other *streptococcus* spp. (n, %) | 41 (17.2) | 5 (38.5) | 36 (16) | 0.053 | 0.248 (0.084, 0.728) |
| Time to active antibiotics, hours (Median, IQR) | 1.9 (0.2, 5.5) | 3.0 (2.6, 4.0) | 1.7 (0.2, 5.5) | 0.312 |  |
